# Supplementary material for: MetaRibo-Seq measures translation in microbiomes
Source: Nat Commun. 2020 Jun 29;11:3268. doi: 10.1038/s41467-020-17081-z (PMC7324362; doi:10.1038/s41467-020-17081-z)
Supplement: Supplementary file 10 — Supplementary Data 7 [file 41467_2020_17081_MOESM10_ESM.zip › File2/Confidence_High_Taxonomy/108683_out.krona.html]

Javascript must be enabled to view this page.

members
magnitude
magnitudeUnassigned
count
unassigned
taxon
rank

108683\_out

19

superkingdom
19
2

phylum
19
1239

186801
19
class

order
19
186802

186803
19
family

19
genus
841

360807

SRS012273\_contig\_number\_contig-100\_10883.131989SRS015694\_contig\_number\_5399SRS015782\_contig\_number\_47302SRS016989\_contig\_number\_contig-100\_5311.68496SRS018351\_contig\_number\_contig-100\_6068.6068SRS018623\_contig\_number\_contig-100\_210.132339SRS022071\_contig\_number\_contig-100\_478.232765SRS045713\_contig\_number\_3705SRS050925\_contig\_number\_36024SRS050998\_contig\_number\_contig-100\_334.97230SRS056259\_contig\_number\_22960SRS056273\_contig\_number\_27774SRS062654\_contig\_number\_21592SRS077194\_contig\_number\_32075SRS098644\_contig\_number\_35900SRS1055076\_contig\_number\_contig-100\_6579.66863SRS144362\_contig\_number\_contig-100\_394.249373SRS893259\_contig\_number\_7014SRS893288\_contig\_number\_1137
species
19
